# One of the Largest Pancreatic Pseudocysts in the Literature: A Case Report

**DOI:** 10.7759/cureus.1493

**Published:** 2017-07-20

**Authors:** Sulaiman Alhassan, Shifa Umar, Mark Lega

**Affiliations:** 1 Division of Pulmonary and Critical Care Medicine, Allegheny General Hospital, Pittsburgh, USA; 2 Department of Medicine, Allegheny General Hospital, Pittsburgh, USA

**Keywords:** pancreatic pseudocyst, pancreatitis

## Abstract

The pancreatic pseudocyst is a pancreatic fluid collection which classically develops due to acute or chronic pancreatitis. A 68-year-old male with the remote history of alcohol abuse presented with abdominal pain secondary to acute pancreatitis. The first computed tomography (CT) of the abdomen showed acute necrotizing pancreatitis. He was initially treated conservatively. Repeat CT of the abdomen after two weeks revealed a peripancreatic fluid collection of 20x12x10 cm. One month later, he became septic following biliary stent placement. Repeat CT of the abdomen showed an enlarging pseudocyst of 25x20x14 cm (estimated 7000 mL of fluid). Percutaneous CT-guided cyst drainage was performed and only three liters of infected fluid could be drained which eventually grew Enterococcus faecalis. Due to lack of improvement, he underwent laparotomy with pancreatic necrosectomy, pseudocyst debridement, and cholecystectomy. The patient did well postoperatively and until one-year follow-up visit. The largest pancreatic pseudocyst in the literature (about 9500 mL) was reported in 1882. To our knowledge, this case is the second largest pseudocyst in the literature which was successfully managed by surgical resection.

## Introduction

The pancreatic pseudocyst is a pancreatic fluid collection which classically develops four weeks after acute pancreatitis, with an approximate incidence rate of 5-15% [1-3]. It can also result from chronic pancreatitis and pancreatic trauma. The highest incidence of pseudocysts was reported in patients with alcohol-related chronic pancreatitis [3]. Literature review reveals very few cases of large pancreatic pseudocysts. We are presenting a case of a giant pancreatic pseudocyst following necrotizing pancreatitis. Informed consent statement was obtained for this study.

## Case presentation

A 68-year-old Caucasian male with a remote history of heavy alcohol abuse who presented with acute abdominal pain and vomiting secondary to acute pancreatitis. The initial computed tomography (CT) of the abdomen showed acute necrotizing pancreatitis with no loculated peripancreatic fluid collection. The ultrasound of the abdomen showed a sludge-filled gallbladder. The patient was initially treated conservatively with no significant improvement. Follow-up CT of the abdomen after two weeks revealed a peripancreatic fluid collection of 20x12x10 cm (estimated 2400 mL of fluid). The prolonged hospital course was complicated by multi-organ failure, mechanical ventilation use, total parenteral nutrition (TPN) administration, and Candida fungemia. No surgical intervention was needed. 

**Figure 1:**
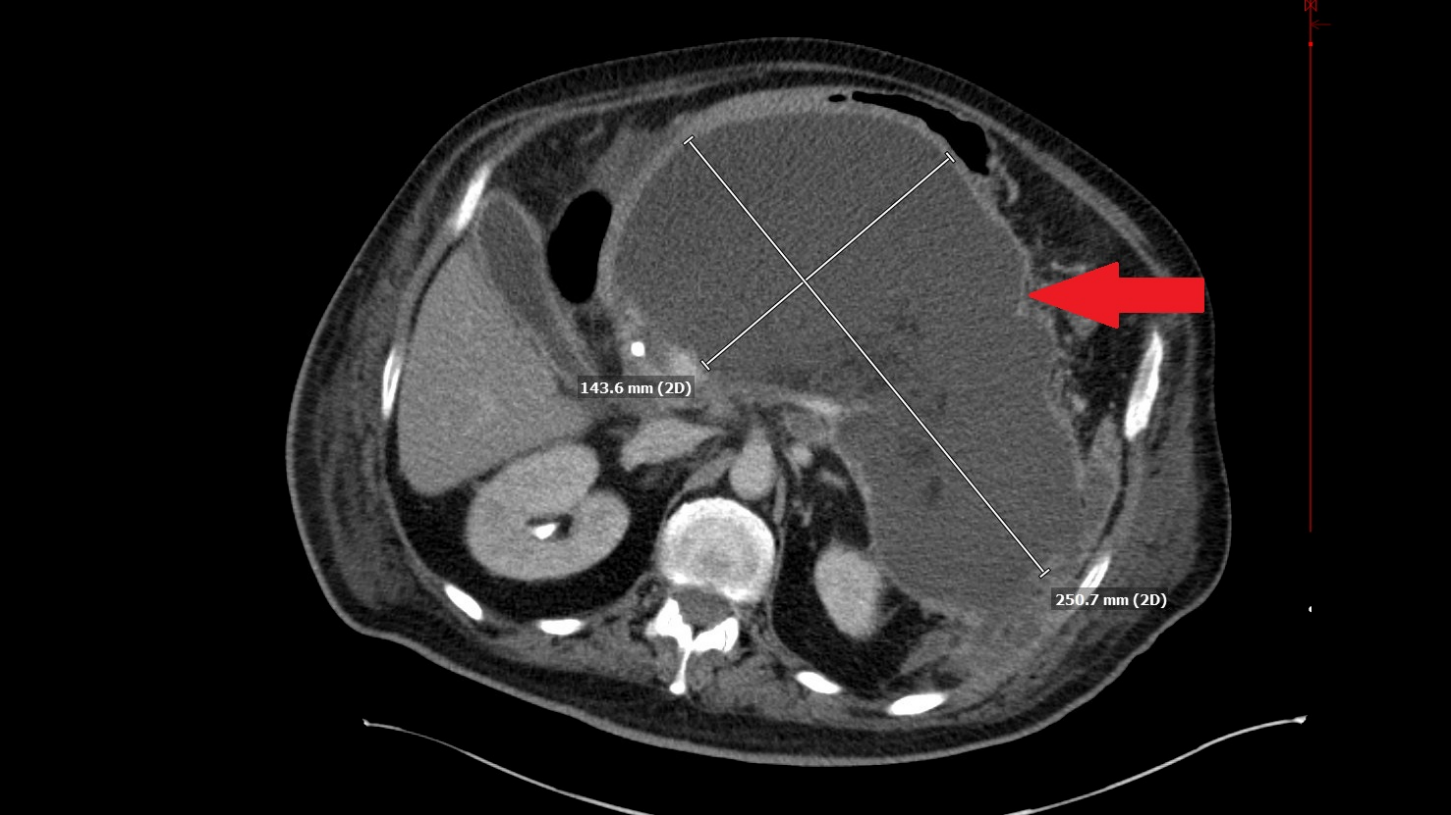
Computed tomography of the abdomen - transverse view Computed tomography of the abdomen showing a transverse view of a large pancreatic pseudocyst

**Figure 2:**
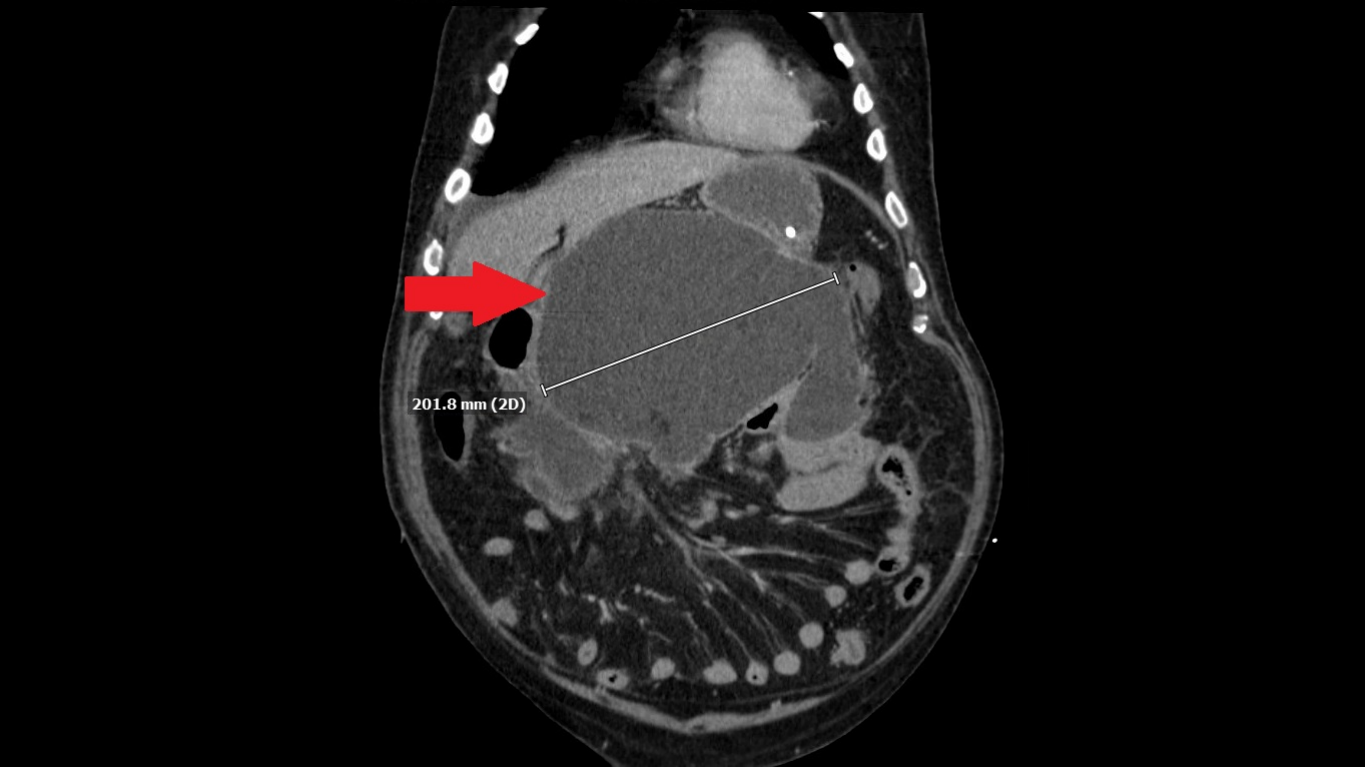
Computed tomography of the abdomen - coronal view Computed tomography of the abdomen showing a coronal view of a large pancreatic pseudocyst

One month later, the patient underwent endoscopic retrograde cholangio-pancreatography (ERCP) with balloon extraction of choledocholithiasis and stent placement. Subsequently, he became encephalopathic and septic. Repeat CT scan of the abdomen showed marked interval increase in the size of the pancreatic pseudocyst to 25x20x14 cm (estimated 7000 mL of fluid) (Figure 1-2). Percutaneous CT-guided cyst drainage was performed and Enterococcus faecalis was isolated from the pancreatic fluid. Only three liters of the infected fluid was able to be drained by the percutaneous drainage. The patient continued to deteriorate in spite of broad-spectrum antibiotics. Thus, he underwent laparotomy with pancreatic necrosectomy, pseudocyst debridement, and cholecystectomy. The patient did well postoperatively and was discharged from the hospital. After one-year follow-up, he is still doing well with no complaints. He has a good oral intake with using pancreatic enzyme replacement. At one-year follow-up, CT of the abdomen showed that the body and tail of the pancreas are surgically absent, with a 5.3x4.9x4.6 cm stable residual pseudocyst in the left upper quadrant.

## Discussion

Atlanta classification of acute pancreatitis published in 2013 defined a pancreatic pseudocyst as "an encapsulated collection of fluid with a well defined inflammatory wall usually outside the pancreas with minimal or no necrosis" [4]. Pseudocyst usually needs at least four weeks after onset of acute pancreatitis to mature. This definition was made to differentiate a pseudocyst from an acute peripancreatic fluid collection which indicates the presence of peripancreatic fluid within the first four weeks following acute pancreatitis [4].

Pancreatic pseudocyst might be asymptomatic or associated with upper gastrointestinal symptoms such as pain, nausea, vomiting, and early satiety. In addition, it can result in serious complications such as compression of adjacent organs (stomach, duodenum, common bile duct, pancreatic duct, and large vessels), fluid infection, hemorrhage, and fistula formation [3,5]. Therapeutic strategies may include observation, endoscopic drainage, percutaneous drainage, and surgical interventions based on the clinical presentation. The surgical approach is indicated when complications occur or if cystic neoplasm is suspected [3]. The optimal timing of surgical intervention is not clear, but usually, six weeks observation after acute pancreatitis is recommended to allow for either pseudocyst wall maturation or spontaneous resolution [6].

The term giant pancreatic pseudocyst is traditionally used when the size is greater than 10 cm [7]. However, pseudocysts larger than 20 cm have been rarely reported. The largest pancreatic pseudocyst in the literature was diagnosed by Bozeman, et al. in 1882, with a weight of 20.5 lb. corresponding approximately to 9.5 liters in size [8]. This cyst was, for the first time treated successfully with a surgical intervention. Walker, et al. reviewed 59 cases in 18 years and reported a pseudocyst as large as 6.1 liters approximately [7,9]. To our knowledge, this case is the second largest pseudocyst in the literature with estimated size of seven liters, which was also successfully managed by surgical resection. 

## Conclusions

The pancreatic pseudocyst is one of the consequences of pancreatitis. It can result in serious complications which may require surgical intervention. Serial abdominal imaging is an important tool to monitor these cysts. Spontaneous resolution may occur, but pseudocyst may continue growing in size in some cases. This case is reporting the second largest pancreatic pseudocyst in the literature after the cyst that was found in 1882. 
